# Supplementary material for: IGF2BP3 as a Novel Prognostic Biomarker and Therapeutic Target in Lung Adenocarcinoma
Source: Cells. 2025 Aug 7;14(15):1222. doi: 10.3390/cells14151222 (PMC12346309; doi:10.3390/cells14151222)
Supplement: Supplementary file 1 [file cells-14-01222-s001.zip › cells-3766997-supplementary/supplementary file s1.pdf]

## Supplementary Figures

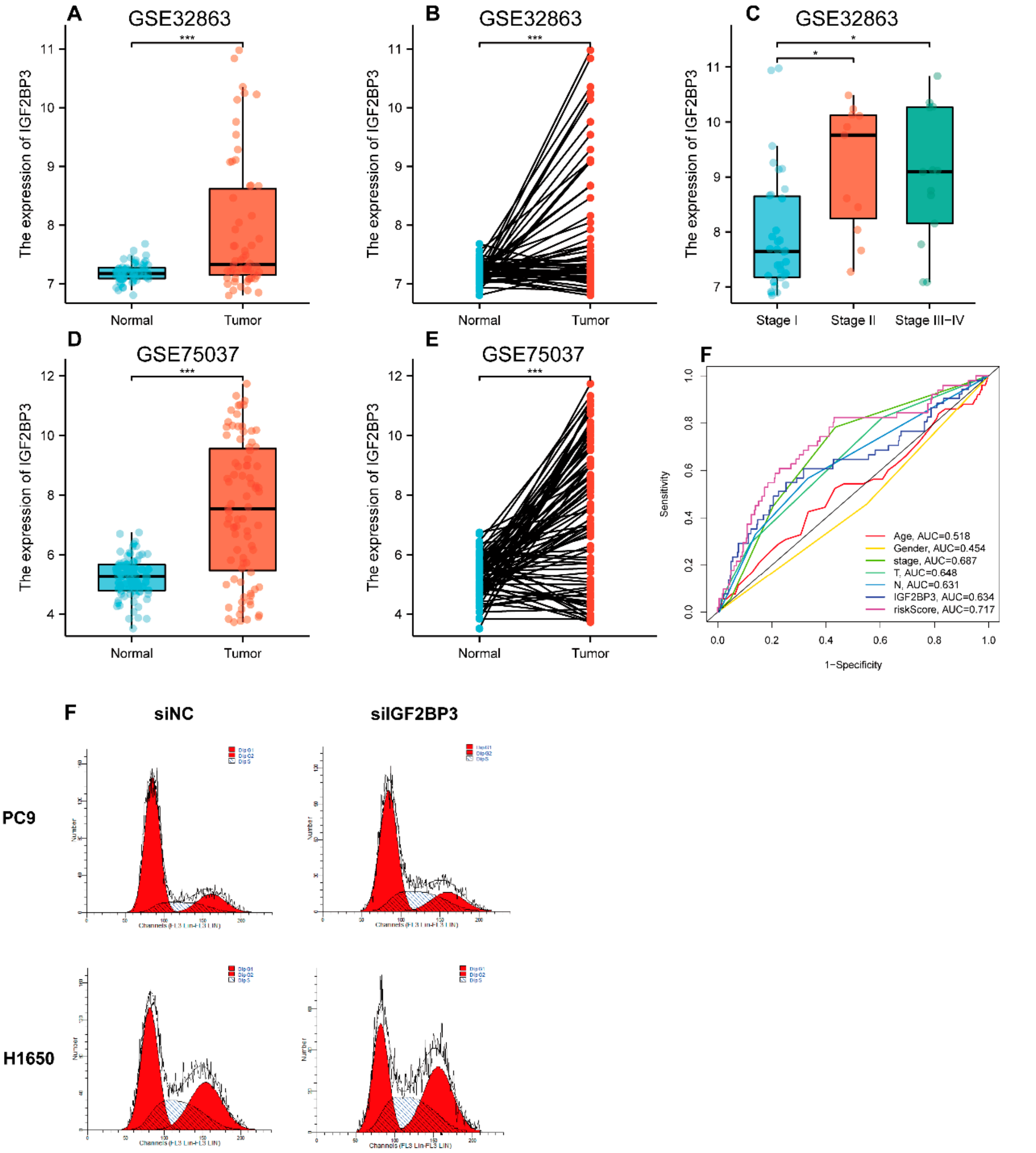


Supplementary Figure S1. IGF2BP3 Expression and Functional Analysis. (A) IGF2BP3 expression across all normal and tumor samples in the GSE32863 dataset. (B) Expression of IGF2BP3 in paired normal and tumor samples from the same patients in the GSE32863 dataset. (C) Expression levels of IGF2BP3 across different stages of disease progression. (D) IGF2BP3 expression across all normal and tumor samples in the GSE75037 dataset. (E) Expression of IGF2BP3 in paired normal and tumor samples from the same patients in the GSE75037 dataset. (F) PC9 and H1650 cells transfected with IGF2BP3 siRNA were subjected to flow cytometry analysis to measure cell cycle distribution. *$P < 0.05$, ***$P < 0.001$.

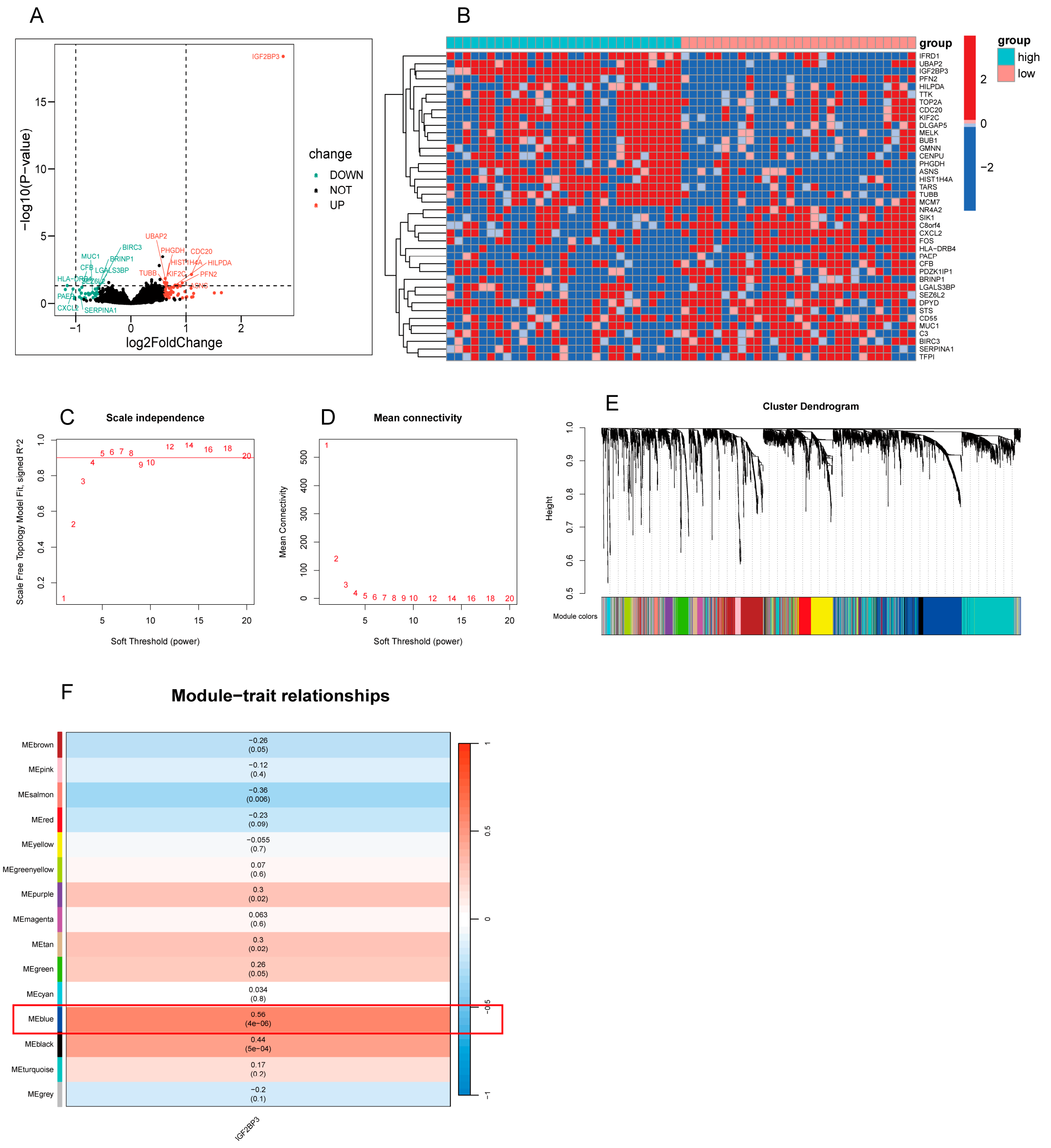


Supplementary Figure S2. Differential gene expression analysis between high and low IGF2BP3 expression groups. (A) Volcano plot of differentially expressed genes (DEGs) in LUAD between high and low IGF2BP3 expression groups, filtered by |log2FoldChange| > 1 and adjusted *p*-value < 0.05. (B) Heatmap representing the differential expression of DEGs between high and low IGF2BP3 expression groups. (C) Scale-free topology fit index calculations across various soft threshold powers (β). (D) Analysis of mean connectivity for different soft threshold powers (β). (E) Hierarchical clustering dendrogram illustrating gene expression modules, with each color representing a unique module. (F) Pearson correlation analysis between module eigengenes and IGF2BP3 expression levels.

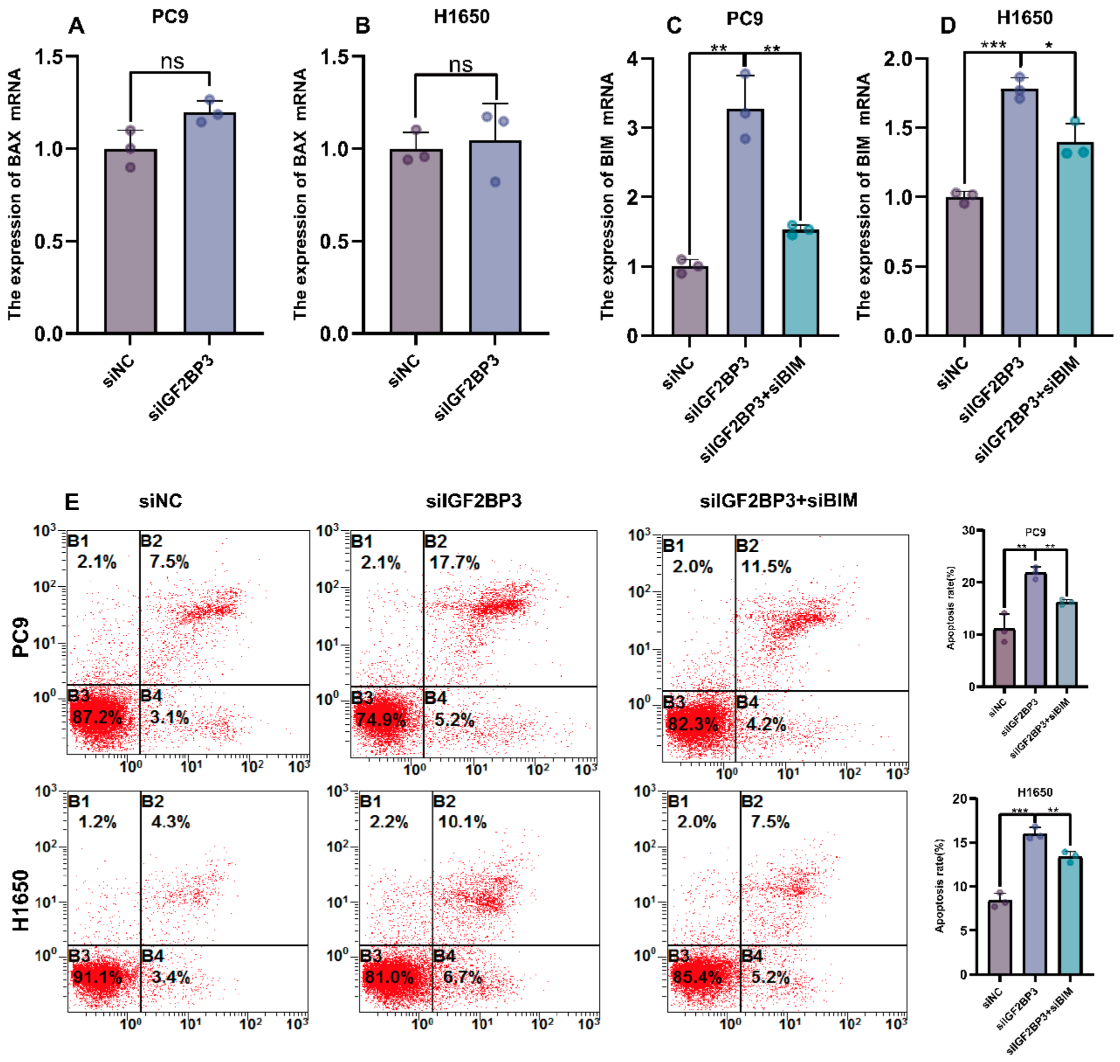


Supplementary Figure S3. siBIM partially counteracted the increased apoptosis in cancer cells caused by IGF2BP3 knockdown. (A-B) The expression levels of apoptosis-related gene BAX were analyzed by qRT-PCR and Western blot methods after knockdown of IGF2BP3. (C-D) The mRNA expression level of BIM was assessed by transfection of siIGF2BP3 and siBIM using plasmids using qRT-PCR. (E) Flow cytometry was performed to detect the number of apoptotic cells co-transfected with siIGF2BP3 and siBIM against PC9 and H1650. **P* < 0.05, ***P* < 0.01, ****P* < 0.001; ns: not significant.

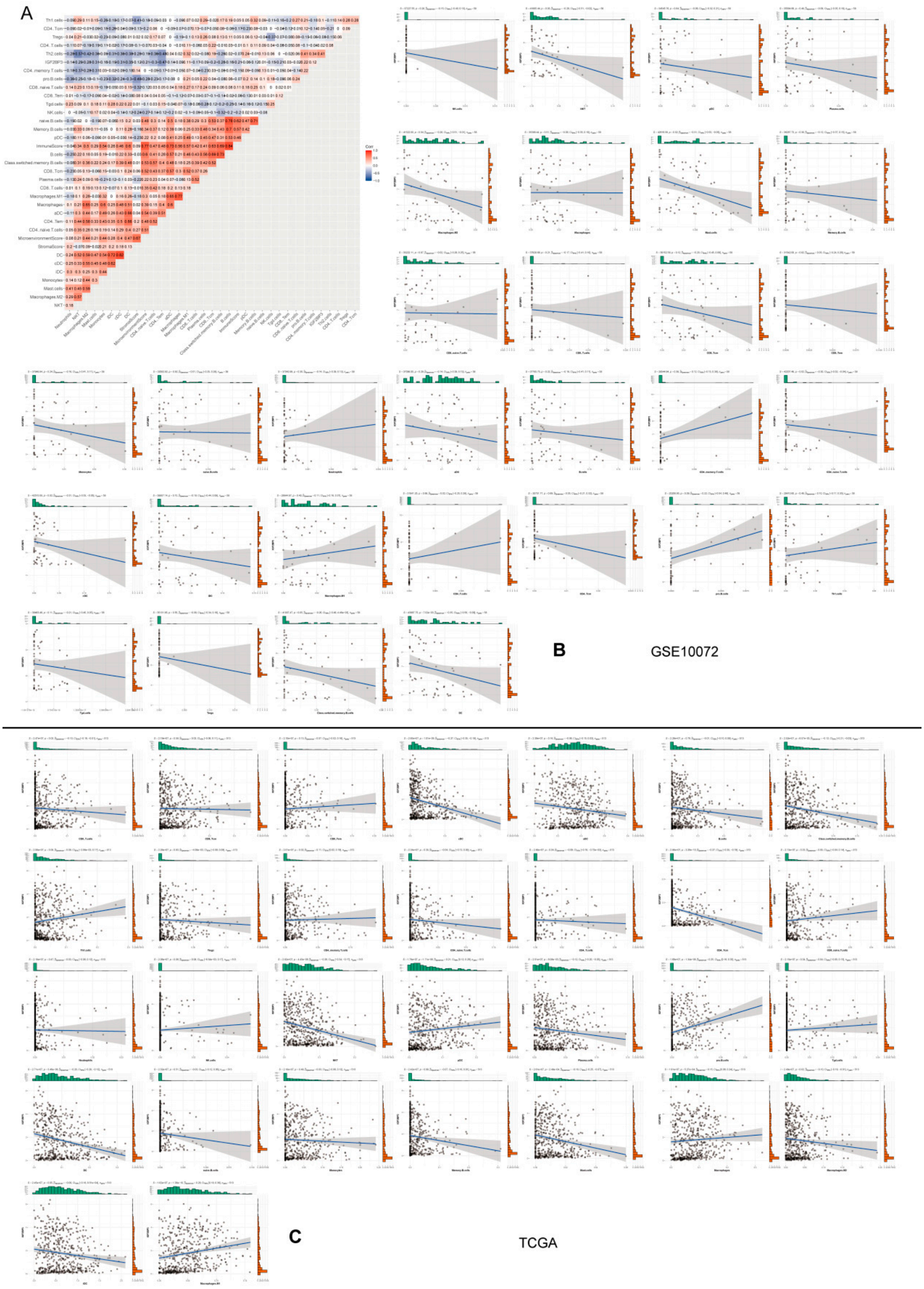


Supplementary Figure S4. Correlation analysis between IGF2BP3 expression and tumor-infiltrating immune cells (TICs). (A) Heatmap depicting the correlation between tumor-infiltrating lymphocytes and IGF2BP3 expression in the GSE10072 dataset, where each color square represents the correlation value between the two cell types. (B–C) Scatter plots showing the correlation between the expression levels of TICs and IGF2BP3 expression levels. The blue line in each plot fits a linear model, and the correlation is tested using Pearson's

coefficient.
