# Supplementary material for: IGF2BP3 as a Novel Prognostic Biomarker and Therapeutic Target in Lung Adenocarcinoma
Source: Cells. 2025 Aug 7;14(15):1222. doi: 10.3390/cells14151222 (PMC12346309; doi:10.3390/cells14151222)
Supplement: Supplementary file 1 [file cells-14-01222-s001.zip › cells-3766997-supplementary/supplementary file s2.pdf]

Supplementary Table S1. Primer sequence

	Forward primer (5′→3′)	Reverse primer (5′→3′)
IGF2BP3	TATATCGGAAACCTCAGCGAGA	GGACCGAGTGCTCAACTTCT
BIM	TAAGTTCTGAGTGTGACCGA	GCTCTGTCTGTAGGGAGGTA
BCL-2	GGTGGGGTCATGTGTGTGG	CGGTTCAGGTACTCAGTCAT
Actin	CATGTACGTTGCTATCCAGGC	CTCCTTAATGTCACGCACGAT

Supplementary Table S2. Primary and secondary antibodies and dilution ratio

Target	Company	Cat.No.	Dilution ratio
IGF2BP3	Proteintech,China	CY6812	WB 1:5000
β-actin	Proteintech,China	66009-1-Ig	1:3000
BIM	Abways,China	CY5307	1:2000
BCL-2	Abways,China	CY5032	1:1500
anti-mouse HRP secondary antibody	Immunoway,USA	RS0001	1:3000
anti-rabbit HRP secondary antibody	Immunoway,USA	RS0002	1:3000

Supplementary Table S3. The sequence of siRNA

Plasmids	sequences (5' > 3')
siIGF2BP3	UUCCUGCAAUGGAGAUAUCTT
